# Two New Phenols from *Scutellaria barbata*

**DOI:** 10.3390/molecules16021402

**Published:** 2011-02-07

**Authors:** Gang Wang, Fei Wang, Ji-Kai Liu

**Affiliations:** 1 Anhui Key Laboratory of Modernized Chinese Materia Medica, Anhui University of Traditional Chinese Medicine, Hefei 230031, China; 2 BioBioPha Co., Ltd., Kunming 650204, China; 3 State Key Laboratory of Phytochemistry and Plant Resources in West China, Kunming Institute of Botany, Chinese Academy of Sciences, Kunming 650204, China

**Keywords:** *Scutellaria barbata*, 2(*S*)-2’,7-dihydroxy-5,8-dimethoxyflavanone, (*S*)-2-(4-hydroxyphenyl)-6-methyl-2,3-dihydro-4H-pyran-4-one, Labiatae

## Abstract

Two new phenols, 2(*S*)-2’,7-dihydroxy-5,8-dimethoxyflavanone (**1**) and (*S*)-2-(4-hydroxyphenyl)-6-methyl-2,3-dihydro-4H-pyran-4-one (**2**), were isolated from the ethanol extract of *Scutellaria barbata*. Their structures were elucidated on the basis of spectroscopic analysis.

## 1. Introduction

*Scutellaria barbata* (family Labiatae) and some other species from the same genus have been widely used in China, India, Nepal and other Asian countries for a long time as a traditional Chinese medicine or a folk remedy for the treatment of different diseases [1,2], and are also recognized as a source of *neo*-clerodane diterpenoids, as more than 150 *neo*-clerodane diterpenoids have been isolated from this genus [3,4,5,6,7,8,9,10,11,12,13,14,15]. BZL101 (Bezielle) is an aqueous extract from *Scutellaria barbata*. BZL101 is currently in phase II clinical trial in patients with advanced breast cancer. The phase I trial showed favorable toxicity profile and promising efficacy [16,17]. In previous investigation we reported four new *neo*-clerodane diterpenoids and structure revision of a series of 13-spiro *neo*-clerodanes [1]. In current study the flavonoid constituents of this plant have been examined. As described in the experimental section, two new phenols: 2(*S*)-2’,7-dihydroxy-5,8-dimethoxyflavanone (**1**) and (*S*)-2-(4-hydroxyphenyl)-6-methyl-2,3-dihydro-4H-pyran-4-one (**2**), were isolated, together with seven known compounds, from the ethanol extract of whole plant of this plant, which was collected in Yunnan province, in southwest China.

## 2. Results and Discussion

**Figure 1:**
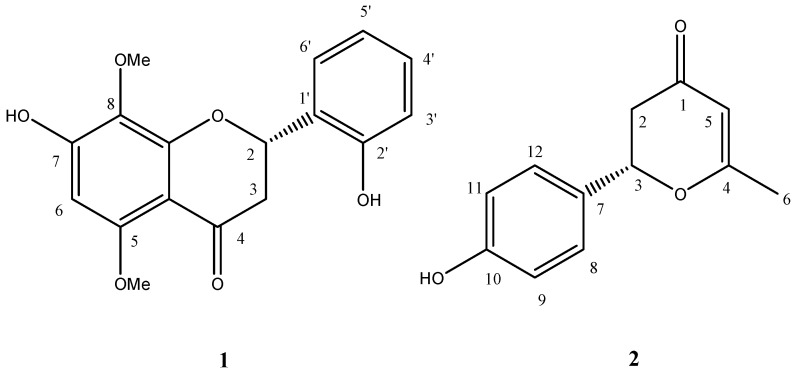
Structures of compounds **1** and **2**.

Compound **1**, obtained as an amorphous powder, had a molecular formula of C_17_H_16_O_6_ based on the positive high resolution electrospray ionization (ESI-MS), showing a quasi-molecular ion peak at *m/z* 317.1024 (calcd. for C_17_H_17_O_6_, 317.1025). The IR spectrum gave absorption bands corresponding to hydroxyl and conjugated carbonyl groups and aromatic rings. The UV spectrum was characteristic of the flavanone series. The flavanone nucleus was also confirmed by ^1^H-NMR spectrum, in which the signals due to the C-3 and C-2 protons were observed an ABX system at 2.58 (1H, dd, 2.8 and 16.4 Hz), 2.92 (1H, dd, *J* = 12.8 and 16.4 Hz) and 5.61 (1H, dd, *J* = 2.8 and 12.8 Hz) [2]. The ^1^H-NMR spectrum further showed the presence of two methoxyls (3.62, 3.69 ppm), two hydroxyls (9.91, 10.45 ppm, no chelated hydroxyl). In the aromatic region of the spectrum, the remaining five protons occurred as a singlet (6.13 ppm, 1H), for the A-ring proton and two doublets (7.43 ppm, 1H, *J* = 7.0 Hz; 6.86 ppm, 1H, *J* = 7.7 Hz) and two multiplets (centered at 6.87 ppm, 1H; 7.18 ppm, 1H) for the B-ring protons [2]. The HMBC correlations between H-6 (6.13 ppm), H-2 (5.61 ppm), H-3a (2.92 ppm), and H-3b (2.58 ppm) and carbonyl (188.5 ppm), 2’-OH (9.91 ppm) and C-2’ (154.4 ppm), C-5 OMe (3.69 ppm) and C-5 (157.3 ppm), C-8 OMe (3.62 ppm) and C-8 (129.2 ppm), 7-OH (10.45) and C-6 (93.3 ppm), C-7 (157.1 ppm) and C-8 (129.2 ppm), 2’-OH (9.91 ppm) and C-1’ (125.4), C-2’ (154.4 ppm) and C-3’(115.5 ppm) were observed. It allowed us to position the two methoxyls in the A-ring at the C-5 and C-8. The chemical shifts and splitting patterns of the B-ring protons suggested that the B-ring is substituted at the 2’-position by a hydroxyl. The arrangement of the substituent in the B-ring was also supported by ^13^C-NMR spectrum. It is known that flavanones having 2(*S*)-configuration exhibit a positive Cotton effect due to n-p* transition (~ 330 nm) and a negative Cotton effect due to p-p* transition (270-290 nm) in the circular dichroism (CD) spectra [18]. The CD curve of **1** exhibited positive and negative maxima at 330 and 285 nm, respectively, which established the 2-(*S*)-configuration. From these results, the structure of **1** was determined to be 2(*S*)-2’,7-dihydroxy-5,8-dimethoxyflavanone (Figure 1). 

Compound **2** was obtained as an amorphous powder. It had a molecular formula of C_12_H_12_O_3_ based on the positive high resolution electrospray ionization (ESI-MS), showing a quasi-molecular ion peak at *m/z* 227.0683 (calcd. for C_12_H_12_O_3_Na, 227.0684). The ^13^C-NMR spectrum revealed 12 carbon resonances, including one carbonyl carbon at d_C_ 191.8, one trisubstituted double bond at d_C_ 174.0 (quaternary carbon) and 104.4 (CH), aromatic carbons at d_C_ 157.7 (quaternary carbon), 128.7 (quaternary carbon), 128.3 (2×CH), 115.2 (2×CH), one oxygen-bearing carbon at d_C_ 80.2 (CH), one methylene carbon at d_C_ 41.4 (CH_2_), and one methyl at d_C_ 20.6 (CH_3_). The ^1^H-NMR spectrum further showed the presence of one methoxyl (1.99 ppm), one hydroxyl (9.61 ppm), one methylene (2.36 ppm, dd, *J* = 3.4, 16.6 Hz; 2.83 ppm, dd, *J* = 14, 16.6 Hz), one proton for the double bond (5.34 ppm, s, 1H), one methine (5.36 ppm, m, 1H). In the aromatic region of the spectrum, the four protons occurred as two doublets (7.27 ppm, 2H, *J* = 7.5 Hz; 6.76 ppm, 2H, *J* = 7.5 Hz). The HMBC correlations between H-5 (5.34 ppm), H-2 (2.83, 2.36 ppm) and carbonyl (191.8 ppm), 10-OH (9.61 ppm), H-9, 11 (6.76 ppm), H-8, 12 (7.27 ppm) and C-10 (157.7 ppm), H-9, 11 (6.76 ppm), H-8, 12 (7.27 ppm), H-3 (5.36 ppm) and C-7 (128.7 ppm), 10-OH (9.61 ppm), H-9, 11 (6.76 ppm), H-8, 12 (7.27 ppm) and C-9, 11 (115.2 ppm), H-8, 12 (7.27 ppm), H-2a (2.83 ppm) and C-3 (80.2 ppm), H-5 (5.34 ppm) and C-2 (41.4 ppm) and C-6 (20.6 ppm) were observed. The optical rotation of compound **2** is -21.7, which established the (*S*)-configuration by data comparison with literature [19]. Thus the structure of **2** was established as (*S*)-2-(4-hydroxyphenyl)-6-methyl-2,3-dihydro-4H-pyran-4-one (Figure 1). It was isolated as a new natural product in present study, and synthesized in a previous report [20].

Compounds **3**-**9** were known compounds and were identified as 2’,4’-dihydroxy-2,3’,6’-trimethoxychalcone [2], apigenin 5-O-beta-D-glucopyranoside [21], 4’,5- dihydroxy-3’,5’,6,7-tetramethoxyflavone [22], 4’-hydroxywogonin [23], 6-methoxynaringenin [24], 2’,5,7-trihydroxy-8-methoxyflavanone [25], 7-hydroxy-2’,5, 8-trimethoxyflavanone [2], respectively, by comparison of their spectral data with literature data.

## 3. Experimental

### 3.1. General

Optical rotations were measured on a Jasco P-1020 (Jasco International Co., Ltd., Tokyo, Japan) automatic digital polarimeter. UV spectra were obtained using a Shimadzu UV-2401A spectrophotometer. IR spectra were recorded using a Bruker Tensor 27 FT-IR (Bruker Optics GmbH, Ettlingen, Germany) spectrophotometer with KBr pellets. NMR spectra were carried out on either a Bruker DRX-500 or AM-400 (Bruker BioSpin GmbH, Rheinstetten, Germany) spectrometers with the deuterated solvent as an internal standard. ESI-MS (including HR-ESI-MS) were performed on an API-Qstar-Pulsar i (MDS Sciex, Concord, ON, Canada) mass spectrometer. Column chromatography was performed on Silica gel (200-300 mesh, Qingdao Marine Chemical Inc., Qingdao, China) and RP-18 (20–45 µm, Fuji Silysia Chemical Ltd., Kasugai, Achi, Japan). Fractions were monitored by TLC (GF 254, Qingdao Marine Chemical Inc., Qingdao, China), and spots were visualized by heating silica gel plates sprayed with 10 % H_2_SO_4_ in EtOH.

### 3.2. Plant material

The whole plants of *S. barbata* were collected in Xinping County of Yunnan Province, China in March 2008, and identified by Mr. Yu Chen of Kunming Institute of Botany, CAS. A voucher specimen (No. BBP2010010SB) was deposited at BioBioPha.

### 3.3. Extraction and isolation

Dried and powdered *S. barbata* whole plants (9,500 g) were extracted three times with 95% EtOH (60 L) at room temperature for two days each time. The extract was concentrated to give a residue (1,000 g), which was fractionalized by silica gel column chromatography eluted with a solvent system of petroleum ether (PE)/acetone (20:1, 10:1, 6:1, 3:1, 1:1, 0:1) and then pure methanol to yield fractions A-G. Fraction D eluted by 33% acetone was separated on silica gel using a solvent system of CHCl_3_/MeOH (30:1→8:1) to obtain subfraction, it was further isolated and purified by silica gel, Sephadex LH-20 (CHCl_3_/MeOH, 1:1) and MCI (50%→100% MeOH in water) columns to afford compounds **5** (46 mg), and **9** (235 mg). Fraction E eluted by 50% acetone was separated on silica gel using a solvent system of CHCl_3_/MeOH (30:1→8:1) to obtain subfractions I and II. Subfraction I was further isolated and purified by silica gel, Sephadex LH-20 (CHCl_3_/MeOH, 1:1) and MCI (50%→100% MeOH in water) columns to afford compounds **1** (67 mg), **2** (70 mg), **3** (94 mg), **4** (543 mg), **6** (86 mg), **7** (128 mg), **8** (3 mg).

### 3.4. Characterization of compound ***1***

2(*S*)-2’,7-Dihydroxy-5,8-dimethoxyflavanone (**1**), powder; [*α*]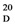 –19.1 (*c* 0.021, MeOH). UV (MeOH): *λ*_max_ (log *ε*) 207 (3.76), 285 (3.02) nm. IR (KBr): *ν*_max_ 3266, 1614, 1583, 1510, 1458, 1416, 1367, 1270, 1099, 992, 748 cm^-1^. ^1^H-NMR (DMSO) and ^13^C NMR (DMSO) data, see Table 1. HRMS [(+)ESI]: *m/z* 317.1024 (calcd. 317.1025 for C_17_H_17_O_6_, [M + H]^+^).

### 3.5. Characterization of compound ***2***

(*S*)-2-(4-Hydroxyphenyl)-6-methyl-2,3-dihydro-4H-pyran-4-one (**2**), powder; [*α*]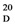 –21.7 (*c* 0.030, MeOH). ^1^H NMR (DMSO) and ^13^C NMR (DMSO) data, see Table 1. HRMS [(+)ESI]: *m/z* 227.0683 (calcd. 227.0684 for C_12_H_12_O_3_Na [M + Na]^+^).

**Table 1:** ^1^H and ^13^C NMR data of **1** and **2** in DMSO (*δ* in ppm, *J* in Hz).

Position	1			2	
	*δ*_H_	*δ*_C_		*δ*_H_	*δ*_C_
1					191.8 (C)
2	5.61 (dd, 2.8, 12.8)	74.1 (CH)		2.36 (dd, 3.4, 16.6)	41.4 (CH_2_)
				2.83 (dd, 14, 16.6)	
3a	2.58 (dd, 2.8, 16.4)	43.8 (CH_2_)		5.36 (m)	80.2 (CH)
3b	2.92 (dd, 12.8,16.4)				
4		188.5 (C)			174.0 (C)
4a		104.7 (C)			
5		157.3 (C)		5.34 (s)	104.4 (CH)
6	6.13 (s)	93.3 (CH)		1.99 (s)	20.6 (CH_3_)
7		157.1 (C)			128.7 (C)
8		129.2 (C)		7.27 (d, 7.5)	128.3 (CH)
8a		156.9 (C)			
9				6.76 (d, 7.5)	115.2 (CH)
10					157.7 (C)
11				6.76 (d, 7.5)	115.2 (CH)
12				7.27 (d, 7.5)	128.3 (CH)
1′		125.4 (C)			
2′		154.4 (C)			
3′	6.86 (d, 7.7)	115.5 (CH)			
4’	7.18 (m)	129.4 (CH)			
5’	6.87 (m)	119.4 (CH)			
6’	7.43 (d, 7.0)	126.8 (CH)			
5-OCH_3_	3.69 (s)	55.8 (CH_3_)			
8-OCH_3_	3.62 (s)	60.6 (CH_3_)			
7-OH	10.45 (s)				
2’-OH	9.91 (s)				
10-OH				9.61(s)	

## 4. Conclusions

In conclusion, two new phenols, 2(*S*)- 2’,7-dihydroxy-5,8-dimethoxyflavanone (**1**) and (*S*)-2-(4-hydroxyphenyl)-6-methyl-2,3-dihydro-4H-pyran-4-one (**2**), were isolated from the ethanol extract of *Scutellaria barbata*. The discovery of compounds **1**-**2** is a further addition to the diverse plant phenolic compounds. Their biological activities are evaluating in progress. 

## References

[1] Wang F., Ren F.C., Li Y.J., Liu J.K. (2010). Scutebarbatines W–Z, New *neo*-Clerodane Diterpenoids from *Scutellaria barbata* and Structure Revision of a Series of 13-Spiro *neo*-Clerodane.. Chem. Pharm. Bull..

[2] Tomimori T., Miyaichi Y., Imoto Y., Kizu H., Namba T. (1985). Studies on Nepalese Crude Drugs. V. On the Flavonoid Constituents of the Root of *Scutellaria discolor*. Chem. Pharm. Bull..

[3] Wang Z.Q., Xu F.M., Yan X.Z., Zhu Y. (1996). Scutebarbatine A, a new neoclerodane-type diterpenoid alkaloid from *Scutellaria barbata*. Chin. Chem. Lett..

[4] Dai S.J., Chen M., Liu K., Jiang Y.T., Shen L. (2006). Four New neo-Clerodane Diterpenoid Alkaloids from *Scutellaria barbata* with Cytotoxic Activities. Chem. Pharm. Bull..

[5] Dai S.J., Wang G.F., Chen M., Liu K., Shen L. (2007). Five New neo-Clerodane Diterpenoid Alkaloids from *Scutellaria barbata* with Cytotoxic Activities. Chem. Pharm. Bull..

[6] Dai S.J., Tao J.Y., Liu K., Jiang Y. T., Shen L. (2006). Neo-Clerodane diterpenoids from *Scutellaria barbata* with cytotoxic activities. Phytochemistry.

[7] Dai S.J., Sun J.Y., Ren Y., Liu K., Shen L. (2007). Bioactive ent-Clerodane Diterpenoids from *Scutellaria barbata*. Planta Med..

[8] Dai S.J., Liang D.D., Ren Y., Liu K., Shen L. (2008). New neo-Clerodane diterpenoids from *Scutellaria barbata* with cytotoxic activities. Chem. Pharm. Bull..

[9] Dai S.J., Shen L., Ren Y. (2008). Two New neo-Clerodane Diterpenoid Alkaloids from *Scutellaria barbata*. J. Integr. Plant Biol..

[10] Dai S.J., Peng W.B., Shen L., Zhang D.W., Ren Y. (2009). Two New neo-Clerodane Diterpenoid Alkaloids from *Scutellaria barbata* with cytotoxic activities. J. Asian Nat. Prod. Res..

[11] Dai S.J., Peng W.B., Zhang D.W., Shen L., Wang W.Y., Ren Y. (2009). Cytotoxic neo-Clerodane Diterpenoid Alkaloids from *Scutellaria barbata*. J. Nat. Prod..

[12] Nguyen V.H., Pham V.C., Nguyen T.T.H., Tran V.H., Doan T.M.H. (2009). Novel Antioxidant neo-Clerodane Diterpenoids from *Scutellaria barbata*. Eur. J. Org. Chem..

[13] Lee H., Kim Y.J., Choi I., Min B.S., Shim S.H. (2010). Two novel neo-Clerodane Diterpenoids from *Scutellaria barbata*. Bioorg.Med. Chem. Lett..

[14] Zhu F., Di Y.T., Liu L.L., Zhang Q., Fang X., Yang T.Q., Hao X.J., He H.P. (2010). Cytotoxic Neoclerodane Diterpenoid Alkaloids from *Scutellaria barbata*. J. Nat. Prod..

[15] Dai S.J., Qu G.W., Yu Q.Y., Zhang D.W., Li G.S. (2010). New neo-Clerodane Diterpenoids from *Scutellaria barbata*. Fiterapia.

[16] Fong S., Shoemaker M., Cadaoas J., Lo A., Liao W., Tagliaferri M., Cohen I., Shtivelman E. (2008). Molecular mechanisms underlying selective cytotoxic activity of BZL101, an extract of *Scutellaria barbata*, towards breast cancer cells. Cancer Biol. Ther..

[17] Perez A.T., Arun B., Tripathy D., Tagliaferri M., Shaw H.S., Kimmick G.G., Cohen I., Shtivelman E., Caygill K.A., Grady D., Schactman M., Shapiro C.L. (2010). A phase 1B dose escalation trial of *Scutellaria barbata* (BZL101) for patients with metastatic breast cancer. Breast Cancer Res. Treat..

[18] Gaffield W. (1970). Circular dichroism, optical rotatory dispersion and absolute configuration of flavanones, 3-hydroxyflavanones and their glycosides: Determination of aglycone chirality in flavanone glycosides. Tetrahedron.

[19] Baker-Glenn C., Hodnett N., Reiter M., Ropp S., Ancliff R., Gouverneur V. (2005). A Catalytic Asymmetric Bioorganic Route to Enantioenriched Tetrahedro- and Dihydropyranones. J. Am. Chem. Soc..

[20] Ahmad R., Khera R.A., Villinger A., Langer P. (2009). One-pot synthesis of 6-aryl-2,3-dihydro-4*H*-pyran-4-ones by cyclocondensation of 1,3-diketone dianions with aldehydes. Tetrahedron Lett..

[21] Veit M., Geiger H., Czygan F.C., Markham K.R. (1990). Malonylated flavone 5-*O*-glucosides in the barren sprouts of *Equisetum arvense*. Phytochemiatsy.

[22] Nagao T., Abe F., Kinjo J., Okabe H. (2002). Antiproliferative Constituents in Plants 10. Flavones from the Leaves of *Lantana montevidensis* BRIQ. and Consideration of Structure–Activity Relationship. Biol. Pharm. Bull..

[23] Wang H.Y., Xiao L.H., Liu L., Xu T.X. (2003). Studies on chemical constituents of the roots of *Scutellaria viscidula*. J. Shenyang Pharm. Univ..

[24] Li P., Zhang G.G., Zuo T.T., Wang S.C. (2008). Chemical constituents of the whole plant of *Scutellaria barbata*. J. Shenyang Pharm. Univ..

[25] Miyaichi Y., Imoto Y., Tomimori T., Lin C.C. (1987). Studies on the Constituents of *Scutellaria* Species. IX. On the Flavonoid Constituents of the Root of *Scutellaria indica*. Chem. Pharm. Bull..

